# Nurses’ and midwives’ perspectives on how the pursuit for the ‘perfect’ body image affects their own breastfeeding practices: a qualitative study in Ghana

**DOI:** 10.1186/s13006-021-00421-0

**Published:** 2021-09-26

**Authors:** Angela Kwartemaa Acheampong, Alhassan Sibdow Abukari

**Affiliations:** School of Nursing, Wisconsin International University College-Ghana, Accra, Ghana

**Keywords:** Nurses and midwives, Breastfeeding, Body image and breastfeeding, Body image concerns, Ghana, Sociocultural pressures, And breastfeeding

## Abstract

**Background:**

Body image concerns have been widely documented in the literature as one reason why most women shorten the breastfeeding duration of their infants. Negative body image concerns among breastfeeding mothers may lead to depressive symptoms. There is a paucity of literature on how body image affects the breastfeeding practices of nurses and midwives. Therefore, this study explored the perspectives of breastfeeding nurses and midwives on how their body image affected their breastfeeding practices.

**Methods:**

A qualitative design was used in this study. Five focus group discussions were conducted with each group having five members. The study was conducted in the Greater Accra Region of Ghana between November and December of 2020. Participants were recruited into the study using a purposive sampling method. Focus group sessions were audiotaped and transcribed verbatim. Data were analyzed using a content analysis.

**Results:**

Three main themes emerged from the data analysis: body image concerns and breastfeeding, sociocultural pressures and breastfeeding and coping strategies. Participants had concerns regarding weight gain due to the need to eat adequately while breastfeeding. Body image concerns included increase in abdominal size, sagging breasts and generalized weight gain. These concerns and pressures negatively affected the breastfeeding practices of participants. Body image concerns reflected sociocultural pressures such as negative comments from loved ones and in the social media. The coping strategies adopted by the mothers were self-motivation and the love they had for their children.

**Conclusions:**

The perspectives of breastfeeding nurses and midwives on the ways their body image affected their breastfeeding practices identified the need for support in order to successfully breastfeed.

## Background

The World Health Organization (WHO) recommends that breast milk or human milk should be exclusively provided to infants until 6 months of age and followed with complementary breastfeeding until the infant is 2 years old [1]. The WHO’s directive is based on the myriad of documented positive health outcomes of breastfeeding for the mother, child and society [2–11]. If breastfeeding is practiced globally as recommended, that is, from birth until twenty four months of age, it has the potential to prevent over eight hundred thousand deaths among children under 5 years of age [9]. Regardless of the numerous benefits of breastfeeding, globally, 41% of infants under 6 months were exclusively breastfed between 2013 and 2018 [12]. In Ghana, the rate of exclusive breastfeeding for 5 months dropped by 9.2%, from 52.1% in 2014 to 42.9% in 2018 [13].

Body image concerns have been widely documented in the literature as one reason why most women shorten the breastfeeding duration of their infants [14–24]. Negative body image concerns among breastfeeding mothers may lead to depressive symptoms such as constant feelings of sadness, general disinterest in one’s surroundings, loss of appetite, and reduced self-esteem. These factors may eventually result in a shorter duration of breastfeeding [21, 23, 25, 26]. Body image concerns are also associated with eating disorders among breastfeeding mothers during the postpartum period and may result in maternal obesity [14, 17, 19, 24, 27]. Obesity and overweight concerns may make breastfeeding mothers feel fat and unattractive. Such negative feelings may lead to the early cessation of breastfeeding [16, 20, 24]. During pregnancy some mothers may not intend to breastfeed their infants because of excess weight gain during pregnancy [15, 28]. This situation may be more prevalent when pregnant women are unable to appreciate the changes that occur in the female body during pregnancy [15]. Conversely, in parts of the United Kingdom some breastfeeding mothers view their body image with admiration while breastfeeding [29, 30]. This phenomena may reflect the fact that breastfeeding has been associated with weight loss in young mothers [5, 30].

Some breastfeeding mothers are vulnerable to sociocultural pressure to lose weight immediately after delivery [31]. Unrealistic media representations of women can also add to the pressure to lose weight while breastfeeding [32–34]. Celebrities and some mothers depict weight lost following delivery as automatic and easy; such views may pressure new mothers to lose weight immediately after delivery [31, 33, 35, 36]. Additionally, new mothers are often pressured by partners, family members, colleagues and peers who often have unrealistic expectations that women will lose weight immediately after delivery [31]. Meanwhile, fathers of infants have an important role in supporting and promoting breastfeeding through provision of emotional support and participation in infant feeding decisions [37]. Breastfeeding needs to be considered as a public health issue that is tackled holistically by society [38].

The WHO target is that globally 70% of all infants should be exclusively breastfed for 6 months by the year 2030 [12]. Influential on a mother’s decision to breastfeed is the attitude of health professionals [39]. Nurses and midwives play an important role in mothers’ infant feeding choices and their own breastfeeding practices may affect their advocacy techniques. Currently, there is dearth of studies on breastfeeding practices of nurses and midwives in Ghana. Anecdotally, in modern Ghana formally educated mothers are expected to lose pregnancy weight within the shortest possible period after delivery. Consequently, this study explored the perspectives of breastfeeding nurses and midwives on how their body image affected their breastfeeding practices.

## Methods

### Aim, design and setting

The purpose of this study was to explore the perspectives of breastfeeding nurses and midwives on the ways the pursuit of the ‘perfect’ body image affected their breastfeeding practices. The researchers employed a qualitative exploratory design. The study was conducted in the Greater Accra Region of Ghana. Messages were sent across different nursing and midwifery social media platforms to attract nurses from different hospitals across the Greater Accra Region to participate in the study. This recruitment technique allowed the researchers to interview breastfeeding nurses and midwives across diverse settings. The venues for data collection were selected by the participants and included two public hospitals and one private educational institution.

### Sampling and data collection procedures

The inclusion criterion for this study was breastfeeding nurses and midwives with infants younger than 2 years old. Twenty-five nurses and midwives were purposively recruited through invitations via social media platforms targeting local nursing groups. Participants were aggregated into five focus groups of five members. The interview venues were agreed upon by the participating groups and Covid protocols observed. All participants spoke English during the discussions. A semi-structured interview guide developed specifically for this study included closed-ended questions that elicited the demographic data of the participants. The interview guide was pilot tested among a group of five participants with similar characteristics to the study participants. The results of the pilot study were not included in the results of the main study although the feedback helped the researchers to amend any ambiguous questions. For example, the question “*On what basis do the people in your life because your body image concerns?”* was modified to “*who are the persons in your life who heighten your body image concerns and how do they heighten such concerns?”* The researchers also asked open-ended, non-judgmental questions to encourage spontaneous dialogue among the participants. The discussions were conducted in serene, quiet venues and began with group rules to ensure sustained and active interaction among the group members. The researchers also used probes to elicit thick data from participants. The duration of each group discussion was around hundred and ten minutes. Data saturation was reached during the fifth focus group discussion. At this point the researchers noted that participant responses were similar to those of the previous group discussions. The data were audiotaped and transcribed verbatim.

### Trustworthiness of the study

The same interview guide was used for each focus group discussion. Concurrent data collection and analysis allowed the researchers to probe emerging themes and sub-themes in subsequent discussions. Field notes were taken during the data collection to confirm participants’ perspectives. The data was transcribed verbatim and quotes were used to illustrate the participants’ perspectives, giving them a voice.

### Data analysis

In this study data cleaning was performed initially to remove all identifiable data. Verbatim transcription and data analysis were undertaken concurrently. This strategy ensured emerging themes and sub-themes were probed further. The content analysis followed the steps outlined by Padgett [40]. That is, the data was cleaned, read several times to understand participants’ opinions and codes attached to sentences based on their meanings followed by aggregating of the codes into sub-themes and, finally, into major themes [40]. In order to understand the meaning of the participants’ perspectives the transcripts were read several times before words and phrases were coded. The emergent themes were then discussed by the researchers before identifying the final themes so as to ensure the participants’ perceptions were not distorted. The researchers ensured that they remained non-judgmental and open-minded throughout the data collection and analysis to.

### Ethical considerations

Ethical clearance was obtained from the Institutional Review Board of the 37 Military Hospital (37MH-IRB/NF/IPN/418/2020). Participation was voluntary and written consent obtained prior to participation in the study. The group discussions were audiotaped with the participants’ permission. Participants were informed they could withdraw from the study at any time without any repercussions. Confidentiality was ensured through the allocation of each participant with an alpha-numeric code. The first two letters of each alphanumeric code (FG) represented the phrase; focus group, followed by a number that represented the position (as to whether that discussion was conducted 1st, 2nd 3rd etc.) of that focus group discussion. Finally, the last letter represented the alphabet that was used to symbolize a particular participant within the group. For instance, hypothetically, if a sixth focus group discussion was held, the first person in that group would have been represented as FG6A.

## Results

### Demographic data

Twenty-five health professionals were recruited into the study. The breastfeeding participants included sixteen general nurses, five midwives and four community health nurses. All participants were aged between 26 years and 35 years.

### Themes and sub-themes

The three main themes that emerged from the data were: body image concerns and breastfeeding, sociocultural pressures and breastfeeding, and coping strategies.

### Body image concerns and breastfeeding

This theme identified various concerns shared by breastfeeding nurses and midwives concerning their body shape and image under three sub-themes, namely: concerns over generalized weight gain and breastfeeding; sagging breasts and breastfeeding; and concerns over increased abdominal size and breastfeeding.

### Generalized weight gain and breastfeeding

Participants lamented gaining weight on all parts of their bodies and attributed their weight gain to eating extra food to maintain their milk supply. Most indicated that their response to an excessive weight gain was to switch to a strict dieting regimen which reduced their ability to produce enough breast milk for their infants. Some said they would not practice breastfeeding for the stipulated time because their weight gain prevented them from achieving their desired body image.

*“ . . . sometimes I want to have that figure . . . flat tummy and big back, chest out a little. I was like that oo . . . but breastfeeding makes me eat so I’m unable to maintain my body shape . . . My whole body has increased in size. I really crave for that perfect shape. It would be difficult to practice exclusive breastfeeding for six months and continue with complementary [feeding] till two years because of that.” FG5B*

*“ . . . for me, after giving birth my weight has doubled and sometimes I become very worried* . . . *to produce enough milk, I used to eat a lot . . . Inwardly I complain a lot. I am alarmed. For now, in fact, I am worried deep down, I just want to lose weight and come back to my normal self. Because of that, I am on a strict diet which has negatively affected my breast milk flow.” FG4D*

*“I keep gaining weight on all parts of my body as I eat a lot in order to get enough breast milk for my baby which worries me a lot. Because of that, there is no way I am going to breastfeed till the recommended period.” FG1D*

Conversely, a few of the participants were optimistic over not being worried about their current situation in the hope of resuming their normal body shape when they stopped breastfeeding.

*“I didn’t get sad about it, I saw it as a normal thing as a human being; once you are a woman you will pass through that stage and as the children are growing you won’t eat like the way you were eating and that body image will be restored.” FG1C*

### Sagging breasts and breastfeeding

Some participants indicated that because of sagging breasts as a result of breastfeeding they would not be able to breastfeed for the stipulated time frame.

*“I will not continue to breastfeed my child till two years because my breast would sag.” FG5E*

*“Sometimes, it affects me, last time I stood in front of a mirror and asked myself ‘is that me?’ my breast has totally sagged and sometimes I feel sad. How the shape is, the color, in fact everything has changed about the breast. I cannot breastfeed for that long” FG1D*

### Concerns about increased abdominal size and breastfeeding

Some participants complained about their increased abdominal size and how it made it difficult for them to have the perfect feminine body shape. They attributed their intention not to breastfeed for the recommended period to this concern.

*“After delivery, apart from the stretch marks on my abdomen, the abdomen has turned into a certain funny shape because I cannot be on a strict diet due to breastfeeding. It has become big and not every dress I wear fits . . . If I wear fitting dresses, my top part looks bigger than my down part which should not be the case. A woman must have a perfect feminine shape. Sometimes I look in the mirror and my stomach is bigger than my back. It would be difficult to breastfeed for the recommended period due to that.” FG5A*

*“If this girl is going to breastfeed till the stipulated time frame by WHO, then my abdomen is going to look like something else. It would be difficult.”FG3A*

### Sociocultural pressures and breastfeeding

Under this main theme, participants described how the opinions of individuals in their lives and the pressure of social media influenced their body image concerns.

### Husbands

Most participants reported finding negative comments about their body shape from their husbands were disturbing and pressured them to lose weight in order to feel accepted by their partners. Such pressures led to strict dieting regimens which affected their ability to produce enough breast milk for their infants.

*“Sometimes, my husband makes derogatory comments that, “your breast is finished, everything is gone. Your breast used to be very nice but now, the baby has scattered everything” and sometimes, these comments get to me. Now, because of that, I am on a strict diet and the breast milk flow has reduced” FG1D*

*“I was very slim; with my first baby my weight did not change. But with this, my second baby, I have put on weight and my husband has been complaining. He will tell me that he does not like fat women and that if he wanted one he would have married one. I become uncomfortable anytime he says that and that makes it difficult for me to continue breastfeeding since breastfeeding makes me eat.” FG4E*

“*Yes, the pressure from my husband to lose weight and get the perfect female shape is too much. He makes comments like “you are fat, and your tummy is growing big and won’t you do anything about it?” and because of those comments, I will not breastfeed [for] the recommended period because I have to diet. FG3C*

### Neighbors, colleagues and family members

The pressure to lose weight and get into shape was also received from other people in the lives of the nursing mothers. These people were mostly neighbors, colleagues and family members who made comments such as:

*“One day, when we went outside, a neighbor saw me and said, “When you are pregnant, you’re very fat, after delivery too you’re fat”, and I said, “Are you sure? I think I’m growing lean rather”. And she said, “no . . . no . . . no you’ve grown too fat; it’s not nice” Because of that comment, I’m dieting, and I will not breastfeed them (her twins) too much too.” FG5C*

*“When I started work and I went to my mother’s place, everybody was just complaining, my dad and my aunty. Even just [in the] last three days my aunty saw me for the first time since I gave birth and exclaimed “What is this? Please watch your tummy!” It can be disturbing. It makes me watch my diet critically and the milk does not flow well due to that.” FG2B*

*“They, neighbors and colleagues, keep on complaining, saying “an army nurse and you are growing big like this because of delivery and breastfeeding. You are eating too much and look at your tummy, look at how nice you used to be in your uniform, now can you be in your uniform?” So, those comments make me angry and mostly when I become annoyed too much, my breast milk does not flow.” FG5B*

Conversely, there were a few participants who indicated that they also received negative comments about their bodies but tried not to let such comments to get to them in any way.

*“They do make comments like; ‘your tummy is becoming big and now you look ugly’. Someone told me that; ‘those days, you used to look nicer than this’. So, I was like ‘really!’ I do not allow negative comments to affect me. I believe in myself and that is all.” FG2A*

### Social media

A few participants indicated that ‘before and after’ pictures of celebrities and other ordinary people after a pregnancy sometimes got to them. As a consequence of admiring such pictures they tended to watch their diet which led to a lower production of milk.

*“Social media is now a way of life and sometimes the ‘before and after’ pictures which are posted by some celebrities and other people have a way of getting to me. Anytime I see such pictures, I also attempt to watch what I eat which affects my milk flow.” FG3D*

*“[The] before and after pictures of nursing mothers on Instagram and Facebook sometimes make me reduce my food intake which also affects the amount of breast milk I produce.” FG1E*

A few participants felt that the social media is deceptive and the ‘before and after’ pictures were unrealistic and untrue. They also pointed out that, individuals who post such pictures went for surgeries and later pretended they lost all the weight through exercise and dieting.

*“You should know that there are surgeries that are done by all those people on social media with unrealistic photos for their before and after photo-shoots. But they won’t come out to tell you that they have done surgeries. Therefore, I don’t allow them to get to me” FG1B*

### Coping strategies

This theme describes how participants managed to cope with their body image concerns. Self-motivation and children were the two sub-themes that emerged from the data.

### Self-motivation

Although most participants complained about their body image after pregnancy and while breastfeeding, they coped by reassuring themselves that was a short phase in their lives which was bound to pass and after breastfeeding they would exercise and get back to their desired shape.

*“I encourage myself that when I am done with breastfeeding all my children, I will have to exercise more and reduce my intake of food, that’s what I use to encourage myself.” FG1D*

*“I cannot go out; I have to be breastfeeding the baby at night. My friends will call me for parties at night, but I cannot go out. It was really disturbing me, but I have come to realize that it is still part of life. It is a stage, and I will get out of it eventually.” FG5E*

### Children

Children were the source of hope for the breastfeeding mothers. Although most of the participants had complaints about their body shape, the sight of their children made them forget all concerns they had about their looks:

*“Sometimes when I look at my baby, I get comforted that my body shape may be destroyed because of the obligation to eat in order to produce enough breast milk but my child deserves it.” FG2C*

*“I wish my body image could return to what it used to be but what I have in mind is my child first. I just have to breastfeed my child and forget about my body image although I’m not comfortable with the way I look.” FG1C*

“*My uncle will pick [up] a phone and call me complaining that I should watch my tummy, if not I cannot wear my nursing uniform. At first, I felt really bad, but later anytime I watch my baby I become motivated. So, gradually, I am overcoming their comments.” FG5A*

## Discussion

With regard to their body image concerns, breastfeeding nurses and midwives complained about sagging breasts, size, and shape of their abdomens as well as their general weight gain. Because of their concerns participants reported they did not intend to follow the period of breastfeeding recommended by the WHO. Body image concerns among pregnant and breastfeeding mothers which lead to a shortened breastfeeding duration have been extensively reported in literature [14–23]. Additionally, the intention to initiate and continue breastfeeding are lessened once mothers experience body image concerns [41]. Currently, there is scarcity of literature on breastfeeding health professionals. This situation raises the question of “Who takes care of the caretaker?” Perhaps, society has assumed that once health professionals have extensive knowledge of the health benefits they would automatically breastfeed without any concerns. But health professionals are human beings with the same needs as the rest of us. Consequently, efforts should be made to encourage the formation of mother clubs amongst breastfeeding health professionals so they can encourage each other through the journey of motherhood. Such support may lead to exercise regimen, because positive body image has been reported to increase exercise desires among post-partum women [42]. Additionally, contrary to the perceptions of the participants in this study, it has been reported that breastfeeding often results in weight loss in some postpartum women [5, 43]. This information should be made known to all postpartum women since it may encourage them to breastfeed optimally.

This study has reported sociocultural pressures that directly or indirectly influenced breastfeeding nurses and midwives, such as negative and derogatory comments from husbands. The crucial role of male partners in supporting the initiation and length of breastfeeding duration has been widely reported in literature [37, 44–49]. Anecdotally, in the Ghanaian sociocultural context, modern educated men often prefer their partners to maintain their pre-pregnancy weight following delivery, and this preference may put undue pressure on their breastfeeding partners. Consequently, the partners of breastfeeding mothers need to be educated on the negative impact of their comments on the body shape of their breastfeeding partners. Partners should also be involved at every stage of breastfeeding for them to gain understanding on how to support their spouses. Currently, the most effective intervention to involve fathers in breastfeeding has yet to be identified [50].

The reactions of others in the lives of the participants were also crucial in affecting their breastfeeding practices. Words are powerful and if used without caution may lead to maternal psychological distress. Social media depictions of pregnant and breastfeeding women were also mentioned as a source of pressures for the participants in the current study. For example, ‘before and after’ pregnancy pictures of celebrities on social media were reported by participants as having put pressure on them. Meanwhile, the use of social media to form support groups has been proven to promote breastfeeding [51]. For example, breastfeeding mothers can come together and form social media groups to encourage each other. Therefore, breastfeeding health professionals should be encouraged to use the social media platforms such as WhatsApp, Facebook and Instagram to form breastfeeding support groups to share their experiences and find practical solutions to them.

The participants in this study coped with the challenges of body image concerns. Despite their body image concerns they acknowledged that their infants’ wellbeing and self-motivation helped them to cope. Self-motivation has been reported as one of the stimuli for mothers to cope with the challenges of breastfeeding [52]. Therefore, the authors recommend that, self-motivation techniques should be employed by lactation specialists to motivate breastfeeding mothers to breastfeed successfully.

## Conclusions

This study has highlighted that breastfeeding health professionals, like other mothers, need breastfeeding support. The participants had concerns which need to be addressed if they are to successfully breastfeed. Many had body image concerns following delivery and attributed their concerns to the fact that they could not go on strict diet while breastfeeding. They also felt pressured by their partners and people around them to lose weight and regain the ideal female body shape while breastfeeding. Unrealistic images posted on social media affected some, increasing the pressure to lose weight. Such concerns and pressures affected the participants’ breastfeeding practices to the extent that most did not intend to breastfeed for the recommended time.

The authors recommend that health professionals should seek breastfeeding support, particularly as anecdotally it is mostly assumed that they are knowledgeable about breastfeeding benefits may not need support or special attention. It is also recommended that health facilities should provide dedicated lactation support services to address the needs of breastfeeding health professionals. Future research should investigate the development of clinical guidelines that for clinicians to support breastfeeding health professionals*.*

## Data Availability

The datasets analyzed during this current study are available from the corresponding author on reasonable request.
